# Identification of CCL20 and LCN2 as Efficient Serological Tools for Detection of Hepatocellular Carcinoma

**DOI:** 10.1155/2022/7758735

**Published:** 2022-03-10

**Authors:** Lin Du, Manli Wang, Hui Li, Na Li, Fang Wang

**Affiliations:** ^1^Department of Clinical Laboratory, Xingyi People's Hospital, Xingyi, Guizhou 562400, China; ^2^Department of Clinical Laboratory, Chengdu First People's Hospital, Chengdu, Sichuan 610041, China; ^3^Department of Laboratory Medicine, The First Affiliated Hospital of Xi'an Jiaotong University, Xi'an, Shaanxi 710061, China

## Abstract

**Objectives:**

To discover a more powerful diagnostic tool for the detection of hepatocellular carcinoma (HCC).

**Methods:**

16 extracellularly located candidates were selected by analyzing the expression array datasets in GEO. 10 of them were validated in clinical samples by ELISA. Differences of each variable were compared by one-way ANOVA or Kruskal-Wallis test. CCL20 and LCN2 were determined in all samples (HCC, 167; liver cirrhosis, 106; and healthy control, 106) and finally chosen for the construction of the combination model by binary logistic regression. The models were first built using a comprehensive control, including both liver cirrhosis (LC) and healthy donors. Then, the models were rebuilt by using the LC group alone as a control. ROC analysis was performed to compare the diagnostic efficiency of each indicator.

**Results:**

Levels of CCL20 and LCN2 in HCC sera were significantly higher than those in all controls. Using the comprehensive control, ROC curves showed that the optimum diagnostic cutoff of the CCL20 and LCN2 combination was 0.443 (area under curve (AUC) of 0.927 (95% CI 0.896-0.951), sensitivity of 0.808, specificity of 0.892, and accuracy of 0.859). For detection of HCC from LC control, the optimum diagnostic cutoff was 0.590 (AUC of 0.919 (95% CI 0.880-0.948), sensitivity of 0.814, specificity of 0.868, and accuracy of 0.834). Furthermore, the model maintained diagnostic accuracy for patients with HCC in the early stage, with the sensitivity and specificity of 0.75 and 0.77 from LC control, yet the AFP only reached 0.5 and 0.67, respectively.

**Conclusion:**

A combination model composed of CCL20 and LCN2 may serve as a more efficient tool for distinguishing HCC from nonmalignant liver diseases.

## 1. Introduction

Hepatocellular carcinoma (HCC) is the most common type of primary liver malignancy, accounting for more than 80% of all liver cancers. Recent epidemiological data show that HCC is the fourth leading cause of cancer mortality globally [1]. Over the past 40 years, the 5-year survival for HCC has minimally improved and remains below 20% worldwide [2]. This dismal prognosis is due partly to the lack of reliable approaches for timely diagnosis, resulting in a high proportion of patients being diagnosed at advanced stages. Generally, HCC develops under the settings of liver cirrhosis, which is tightly linked to infections with hepatitis virus (HBV and HCV). Thus, early detection, especially in the high-risk population such as chronic hepatitis and liver cirrhosis, is critical to improve the patients' outcomes [3]. The discovery of novel serum biomarkers with a higher degree of accuracy is a fundamental goal in the early stage screen. Unfortunately, the performance of clinically used biomarkers, such as AFP, AFP-L3, DCP, and GPC3, is far from satisfactory due to the suboptimal sensitivity and specificity [4]. Application of nonprotein markers, including lncRNA, microRNA, and mutated DNA, has been intensively studied in the past decade [5, 6]. However, the most applicable biomarkers for clinical routine surveillance are proteins, which are easily detected (low dependence on operator expertise, no sample pretreatment, and less sample input). Herein, we reported two novel serum biomarkers, CCL20 (C-C motif chemokine ligand 20) and LCN2 (lipocalin 2), which were selected as biomarkers on the ground of mining data from the HCC gene expression array. The secretory characteristics enable them to be easily measured in serum samples. We determined their serum levels in a total of 379 samples and evaluated the diagnostic power of the combination model. The results provide clear evidence for the possible diagnosis of HCC in clinical practice.

## 2. Materials and Methods

### 2.1. Subjects

Consecutive patients with newly diagnosed HCC and live cirrhosis were recruited from Jun. 2019 to Oct. 2020. HCC was detected by ultrasound, CT, or MRI and further confirmed by histopathology. Tumor staging was performed on the basis of the Barcelona Clinic Liver Cancer staging system [7]; HCC at stages 0 and A were classified as early-stage HCC. Patients with liver cirrhosis were diagnosed by color Doppler ultrasound or histopathology; all patients have no evidence of a hepatic mass for at least 3 months before recruitment. The healthy controls were collected from the health examination center of our hospital, with no history of liver disease, no viral hepatitis infection, and no malignant disease. Approval for the study was obtained from the institutional ethics review committee of Xingyi People's Hospital.

### 2.2. Sample Collection, Storage, and ELISA

For each participant, the blood sample was collected at the same time they underwent the first laboratory examination (before any form of medical interventions); the sera were collected by centrifugation and stored at -80 degrees until use. The data of laboratory parameters were extracted from the laboratory information system (LIS). The AFP and CA199 were measured by electrochemiluminescence using the Cobas 8000 e602 Analyzer (Roche Diagnostics, Germany). HBsAg was quantified by the Alinity C electrochemiluminescence analyzer (Abbott, Abbott Park, IL, USA). Serum CCL20, LCN2, SPINK1, MDK, DKK1, SPP1, PODXL, REG3A, LAMC1, and MMP12 were measured by ELISA kits purchased from Boster Biological Technology (Wuhan, China) according to the users' manual.

### 2.3. Statistical Analysis

Normality distribution and homogeneity of variances were conducted for all quantitative variables. Differences between groups were analyzed by one-way ANOVA (for more than 2 groups) or Students' *t*-test (for 2 groups) if the variables passed the assessment. If not, the Kruskal-Wallis test (for more than 2 groups) or Mann-Whitney *U* tests (for 2 groups) were used. Combined diagnostic models were constructed by binary logistic regression using the method of forward LR. Variables of models built using the comprehensive control and the results of Hosmer and Leeshawn tests are listed in Supporting Table 2. The ROC curve analysis was performed to compare the performance of each indicator. The AUC (area under curve) with 95% CI (confidence intervals), sensitivity, specificity, and accuracy were calculated based on ROC curves. The Delong test was applied to compare the differences between the AUC of each curve; the results are shown in Supporting Table 3. All the tests were performed using IBM SPSS software 23, except the Delong test that was performed by MedCalc Software 19.0.4.

## 3. Results

### 3.1. GEO Expression Array Datasets and Biomarker Selection

**Figure 1:**
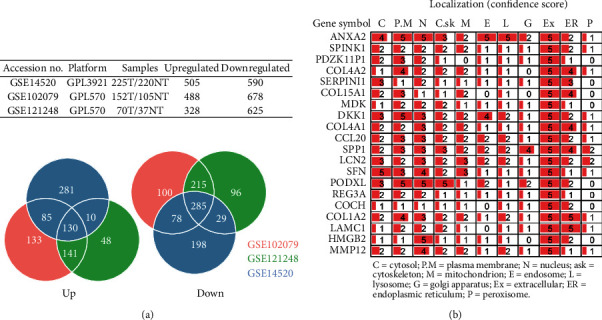
Candidate biomarkers extracted by GEO dataset analysis. (a) Basic information of 3 datasets and the numbers of shared differentially expressed genes. (b) Confidence score of subcellular location of 16 gene products; 5 represents the highest score.

Three datasets [8–10] were selected for HCC differentially expressed genes (DEGs) analysis; numbers of up- and downregulated genes (on the criterion of ∣log fold change | >1 and *p* values < 0.05) for each dataset are listed in Figure 1(a), in which the lower Venn diagrams showed the numbers of shared DEGs. The 130 co-upregulated genes were then subjected to subcellular localization analysis by extracting information from the GeneCards database (http://www.genecards.org); genes with confidence scores equal to 5 were considered potential biomarkers that are present in serum for ELISA. Out of 130, 16 genes met the requirement (Figure 1(b), Supporting Table 1). Using the expression data from GSE14520, we also compared the differences of expression levels between the tumor and nontumor groups (Supporting Figure 1) and analyzed the correlations between each target (Supporting Figure 2).

### 3.2. Baseline Characteristics of Subjects

Serum samples were collected from 379 subjects, containing 106 healthy individuals, 106 LC patients, and 167 patients with newly diagnosed HCC. Both male proportion and age in the HCC group were slightly higher in comparison with the other two. In LC patients, 64.15% were in the compensated stage. In the HCC group, only 5.39% of patients were in the BCLC 0 stage. Nearly half of those were in the late stage (38.32% of BCLC C and 11.98% of BCLC D). For laboratory parameters, data of HBsAg and two tumor biomarkers were collected. In both LC and HCC groups, approximately 90% of subjects were infected with HBV. These data were only collected from the available information; in some LC patients derived from chronic hepatitis B, the HBsAg may test negative for the current samples; thus, the actual figures may be higher. The AFP and CA199 were presented as the median with quartiles for their nonnormal distribution in the LC and HCC groups. In general, both AFP and CA199 showed an upward tendency as the disease progressed, which could be revealed from the increasing medians of each indicator. However, there was actually no significant difference in CA199 levels between the HCC and LC groups (Table 1).

### 3.3. Measurement and Comparison of the Serum Concentrations of 10 Potential Biomarkers

Out of the 16 indicators that are located extracellularly, 10 (CCL20, LCN2, SPINK1, MDK, DKK1, SPP1, PODXL, REG3A, LAMC1, and MMP12) were determined by commercially obtained ELISA kits. For the first step selection, we conducted ELISA in 174 samples (58 of each group). Serum concentrations of CCL20 and LCN2 were remarkably elevated in cancer groups in comparison with noncancer groups. For all the rest indicators, differences between each group were less significant (Supporting Figure 3). The pairwise correlation of these indicators was also analyzed, and the coefficient of 0.29 indicated no correlation between levels of CCL20 and LCN2 (Supporting Figure 4), which was acceptable for both of them in the generation of a combined diagnostic model.

**Figure 2:**
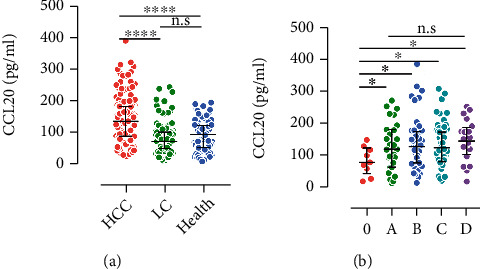
Comparison of level differences: (a) CCL20; (b) LCN2. (a, b) Showed the level differences in HCC group classified by BCLC stage. ^∗^*p* < 0.05,  ^∗∗∗∗^*p* < 0.0001. n.s.: no significance.

We next measured the serum levels of CCL20 and LCN2 in the rest of 205 samples (total, 379). As expected, levels in sera of HCC patients were both significantly higher than the LC and healthy groups (all *p* values < 0.0001) (Figures 2(a) and 2(b)). Levels were then analyzed in HCC subgroups divided by disease stage. An overall increasing tendency along with HCC progression was found for both CCL20 and LCN2 (Figures 2(a) and 2(b)). Pairwise correlation between CCL20, LCN2, AFP, and CA199 was also analyzed, and the results showed that all the coefficients were below 0.3 (Supporting Figure 5), further indicating no correlations between each indicator.

### 3.4. Diagnostic Model Construction and Performance Evaluation

**Figure 3:**
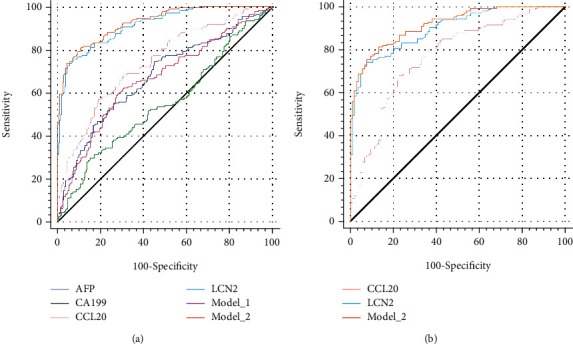
ROC curves indicating methods for diagnosis of HCC using comprehensive controls (healthy+liver cirrhosis) (a) or liver cirrhosis controls (b).

For discriminating HCC using the LC and healthy groups as comprehensive control, three combination models were built. In model_1, the currently used biomarker, AFP, and CA199 were included, while in model_2, CCL20 and LCN2 were used; finally, in model_3, all the four were embodied. Actually, the AFP and CA199 were excluded from model_3, which was identical to model_2 (Supporting Table 2). We next compared the performance of each model in the diagnosis of HCC; besides, the power of CCL20 and LCN2 alone was also examined by ROC analysis (Figure 3(a)).

The diagnostic efficacy of each indicator is revealed in Table 2. At the cutoff of 5.54 and 37.44, AFP and CA199 achieved the AUCs of 0.675 and 0.549, respectively. For CA199, although the specificity was 0.849, only a sensitivity of 0.293 was reached. AFP showed more robust sensitivity (0.743) than CA199, however with the drop of specificity (0.552). For the combination of these two biomarkers, namely, model_1 failed to enhance the discriminating power; the AUC of 0.667 was even lower than that of AFP, although the difference was not significant (*p* = 0.7542, Supporting Table 3). The overall accuracy of 0.662 of model_1 was slightly higher than that of AFP or CA199 alone; however, with the sensitivity and specificity of 0.623 and 0.693, the power of this model was not competent to clinical application. The novel indicators evidently boosted the discriminating power. LCN2 alone could reach an AUC of 0.913, with sensitivity and specificity of 0.743 and 0.943, respectively. When combined with CCL20, the AUC was further increased to 0.927 (*p* = 0.0402 vs. LCN2, Supporting Table 3.). In combination with CCL20, model_2 increased the sensitivity to 0.808 but decreased the specificity to 0.892. The overall accuracy was slightly increased from 0.855 to 0.859. In this regard, we suggested that the combination model_2 was the more acceptable approach.

We rebuilt model_2 by using LC groups as the nontumor disease control (Supporting Table 4), considering that cirrhosis is the principal risk factor for HCC development. ROC analysis was then performed to compare the classification power of the model with indicators used alone (Figure 3(b)).

The results were similar to those using the comprehensive control. LCN2 alone, at the same cutoff, reached a sensitivity, specificity, and overall accuracy of 0.743, 0.925, and 0.812, respectively. The AUC of LCN2 was slightly lower than model_2 (0.898 vs. 0.919, *p* = 0.242). Besides, in comparison with LCN2, relatively higher sensitivity (0.814) and lower specificity (0.834) were revealed by model_2 with an overall accuracy of 0.834 (Table 3).

Finally, the capacity of each indicator for detecting HCC in the early stage defined as BCLC 0 and BCLC A was investigated, for earlier diagnosis was crucial for improving the patients' prognosis. The confusion matrix in Table 4 shows the power of each method mentioned in Table 2 in the classification of early-stage HCC from LC control. Out of 36, CCL20, LCN2, and model_2 identified 18 (0.500), 24 (0.667), and 27 (0.750) cases, respectively. AFP determined half cases (0.500); combined with CA199, only one more case was identified.

## 4. Discussion

Hepatocellular carcinoma, accounting for 85% of primary liver cancers, is one of the most common malignant cancers worldwide. Despite continuous improvement in cancer research and care, HCC remains a major threat to humans, with an increasing global incidence and high associated mortality. The poor prognosis of HCC is largely due to the low diagnosis rate at the early stage, in which no clear symptom is presented. Detection of HCC at late stages precludes timely and eventually curative therapeutic intervention. Patients with HCC in the early stage are eligible for curative treatment and can achieve 5-year survival rates approaching 70% with liver transplantation or surgical resection [11]. Conversely, those with more advanced tumors are only eligible for palliative treatments and have a poor prognosis, with a median survival of 1-2 years [12]. These data emphasize the magnitude of early detection of HCC, which can be achieved by regular surveillance consisting of ultrasound screening every six months. However, less than 40% of patients with cirrhosis undergo proper HCC surveillance [13], and less than 50% of HCC were diagnosed through surveillance [14]. The main cause of low participation in regular surveillance is believed to be the lack of reliable biomarkers with high a high degree of sensitivity and specificity [15]. Serum biomarker is an attractive alternative approach for surveillance and tumor early diagnosis because of its noninvasive and objective characteristics. However, the most commonly used biomarker, AFP, is not recommended in HCC surveillance by the current guideline [16]. On the one hand, nearly half of HCC patients are AFP negative, especially in small HCC, limiting its application largely in advanced HCC [17, 18]. On the other hand, the specificity of AFP is far from satisfactory; elevated levels are frequently found in nontumor diseases of the liver [19]. Some newly introduced biomarkers, such as DCP (des-*γ*-carboxyprothrombin), glycosylated AFP (AFP-L3), *α*-fucosidase, osteopontin (OPN), and GPC-3 (glypican 3), show similar shortcomings. Therefore, it is urgent to discover new indicators to minimize such limitations [20].

In the present study, we examined the serum levels of 10 proteins upregulated in HCC tissues based on GEO datasets; we finally constructed a combined diagnostic model based on CCL20 and LCN2 in HCC detection. CCL20 is a chemokine that is physiologically expressed in multiple tissues and organs, including the liver, colon, and skin. By interacting with the specific receptor, CCR6 (C-C chemokine receptor 6), the CCL20/CCR6 axis is primitively reported to be involved in the regulation of inflammatory response [21]. Recent data also demonstrate its association with cancer progression. In HCC tissue, CCL20 expression is closely related to tumor size and vascular invasion. Patients with high CCL20 levels had poorer recurrence-free survival and overall survival than those with low CCL20 levels [22, 23]. Evidence shows that CCL20 accelerates tumor metastasis through both the induction of EMT (Epithelial-to-Mesenchymal Transition) and inhibition of T-cell proliferation and promoted the expansion of immunosuppressive Treg cells [24]. In brief, CCL20 is harnessed by tumor cells to establish an immunosuppressive tumor microenvironment in favor of its survival and metastasis. Accordingly, CCL20 may serve as an indicator for HCC diagnosis and disease progression. Another molecule we identified that would be used as a diagnostic tool is LCN2, which is a critical iron regulatory protein during physiological and inflammatory conditions [25]. LCN2 is first identified as an acute-phase protein produced by neutrophils during bacterial infections [26]. Given that it is readily detectable in serum or other forms of body fluids, LCN2 has long been investigated as a potential biomarker. It shows promise as a biomarker for the early diagnosis of acute kidney injury and chronic kidney disease [27]. Generally, they are both inflammation-regulatory molecules, which highlight the possibility of non-tumor-specific origination. Herein, we reported the increased serum levels of these two molecules in HCC patients; considering their immune-regulatory roles, HBV infection or liver fibrosis may also drive their overexpression/secretion; we introduced patients with liver cirrhosis for comparison purposes. We found that, for LCN2, serum levels were elevated in cirrhosis patients compared with healthy controls; however, the significance was much lower than that from a comparison between the cirrhosis and HCC groups. Meanwhile, the indicators were selected based on the tumor-specific gene expression profiles. Inflammation in liver tissues may bring about higher CCL20 and LCN2; further production of them by tumor cells would be the main source in HCC sera. Some remaining indicators in our preliminary validation also showed significantly higher serum levels in HCC groups, such as SPINK1, SPP1, and PODXL (Supporting Figure 3); however, for simplifying our model, only the two most significant indicators were further determined in all samples and included in the model. A recent multicenter study revealed that measurement of serum DKK1 has diagnostic value for HCC better than that of AFP, especially for patients with AFP-negative status and early-stage HCC [28]. In our study, DDK1 was one of the candidate indicators according to the GEO data analysis, by which the expression levels of DKK1 in HCC tissues were significantly higher than that in non-HCC tissues (Supporting Figure 1); however, in our first-step validation, we simply found a marginally higher median of DKK1 levels in the HCC population. In comparison with non-HCC groups, the differences were not significant (Supporting Figure 3). The disparity might be linked to the discrepancy in sample size.

We finally evaluated the performance of the CCL20/LCN2-based model (model_2) in discriminating HCC from the comprehensive control that includes all nontumor subjects and the control of patients with liver cirrhosis, which constitutes the highest risk group for the development of HCC. The model revealed a more powerful capacity than did the AFP; especially in the detection of early-stage HCC, model_2 achieved the sensitivity and specificity of 0.75 and 0.77, respectively. Yet, only 0.5 and 0.67 were revealed by AFP. Nevertheless, we realized that we could only collect a small number of early-stage cases in the current study; the diagnostic performance of this model needs to be further explored.

## Figures and Tables

**Table 1:** Baseline characteristics of subjects.

	Healthy (*n* = 106)	LC (*n* = 106)	HCC (*n* = 167)	Sig^a^
Gender (*M*, %)	51.9	52.8	58.7	∗
Age (*M* ± S.E.)	51.56 ± 9.57	52.08 ± 9.44	57.14 ± 10.1	∗
Stage (%)		Compensated: 64.15	BCLC 0: 5.39	
		Decompensated: 35.85	BCLC A: 16.17	
			BCLC B: 28.14	
			BCLC C: 38.32	
			BCLC D: 11.98	
HBsAg (positive, %)		90.6	89.8	n.s.
AFP^b^ (ng/ml)	3.75 (2.09-5.88)	7.81 (3.69-19.74)	9.27 (5.42-31.13)	∗∗
CA199^b^ (ng/ml)	16.36 (9.38-23.54)	18.71 (5.79-41.57)	19.85 (8.36-39.63) ^c^	∗

^a^Comparison between 3 groups. ^b^Data were presented as median with quartile (Q1-Q3). ^c^Not significant vs. LC. LC: liver cirrhosis; HCC: hepatocellular carcinoma; n.s.: not significant. ^∗^*p* < 0.05,  ^∗∗^*p* < 0.01, and^∗∗∗∗^*p* < 0.0001.

**Table 2:** Performance of individual indicators or combination models in the detection of HCC from comprehensive control.

Indicator	Cutoff^a^	AUC^b^	S.E.	CI 95	Sens.	Spec.	Accu.
AFP	5.54	0.675	0.028	0.625-0.722	0.743	0.552	0.636
CA199	37.44	0.549	0.030	0.498-0.600	0.293	0.849	0.604
Model_1	0.416	0.667	0.028	0.617-0.714	0.623	0.693	0.662
CCL20	117.08	0.742	0.025	0.695-0.785	0.569	0.811	0.704
LCN2	94.92	0.913	0.014	0.881-0.940	0.743	0.943	0.855
Model_2	0.443	0.927	0.012	0.896-0.951	0.808	0.892	0.859

AUC: area under curve; S.E.: standard error; CI 95: 95% confidence interval; Sens.: sensitivity; Spec.: specificity; Accu.: accuracy. ^a^Cutoff values were calculated according to maximal Youden index; ^b^differences between AUCs were compared; significance is showed in Supporting Table 3.

**Table 3:** Performance of individual indicators or combination models in detection of HCC from liver cirrhosis control.

Indicator	Cutoff^a^	AUC	S.E.	CI 95	Sens.	Spec.	Accu.
CCL20	94.53	0.772	0.0295	0.718-0.820	0.683	0.774	0.718
LCN2	94.92	0.898 ^b^	0.0179	0.856-0.931	0.743	0.925	0.812
Model_2	0.590	0.919 ^c,d^	0.0155	0.880-0.948	0.814	0.868	0.834

AUC: area under curve; S.E: standard error; CI 95: 95% confidence interval; Sens.: sensitivity; Spec.: specificity; Accu.: accuracy. ^a^Cutoff values were calculated according to maximal Youden index; ^b-d^significance levels of AUC differences; ^b^*p* = 0.0002 vs. CCL20; ^c^*p* < 0.0001 vs. CCL20; ^d^*p* = 0.0242 vs. LCN2.

**Table 4:** Capacity of three to detect early-stage HCC (eHCC).

		Predicted												
		AFP		CA199		Model_1		CCL20		LCN2		Model_2		Total
		eHCC	Ctrl	eHCC	Ctrl	eHCC	Ctrl	eHCC	Ctrl	eHCC	Ctrl	eHCC	Ctrl	
Actual	eHCC	18	18	10	26	19	17	18	18	24	12	27	9	36
	Ctrl	34	72	23	83	20	86	17	89	22	84	24	82	106

## Data Availability

All data included in this study are available upon request by contact with the corresponding author.

## Supplementary Materials

Supplementary Materials Supporting Figure 1: comparison of expression levels of 16 upregulated genes, whose protein products were thought to locate extracellularly with the highest confidence score. Data were derived from GSE14520. ^∗∗∗∗^*p* < 0.0001. Supporting Figure 2: correlation analysis of expression levels of abovementioned genes. Data were derived from GSE14520. Digits in the blocks represented the Pearson correlation coefficients. Supporting Figure 3: comparison of serum concentrations of 10 potential biomarkers for the first-step validation. Each group included 58 samples. ^∗^*p* < 0.05, ^∗∗^*p* < 0.01, ^∗∗∗^*p* < 0.001, and ^∗∗∗∗^*p* < 0.0001. n.s.: not significant. Supporting Figure 4: pairwise correlation analysis of the concentrations between 10 potential biomarkers. Digits in the blocks represented the Pearson correlation coefficients. Supporting Figure 5: pairwise correlation between serum levels of CCL20, LCN2, AFP, and CA199. Data from all samples were used. Digits in the blocks represented the Pearson correlation coefficients. Supporting Table 1: differential expression information and brief description of 16 extracellularly located genes. Supporting Table 2: diagnostic model construction using the comprehensive control (liver cirrhosis+healthy). Supporting Table 3: pairwise comparison of ROC curves. Supporting Table 4: diagnostic model construction using liver cirrhosis as control.
