# Supplementary material for: Identification of CCL20 and LCN2 as Efficient Serological Tools for Detection of Hepatocellular Carcinoma
Source: Dis Markers. 2022 Mar 10;2022:7758735. doi: 10.1155/2022/7758735 (PMC8930252; doi:10.1155/2022/7758735)
Supplement: Supplementary Materials — Supporting Figure 1: comparison of expression levels of 16 upregulated genes, whose protein products were thought to locate extracellularly with the highest confidence score. Data were derived from GSE14520. ∗∗∗∗p < 0.0001. Supporting Figure 2: correlation analysis of expression levels of abovementioned genes. Data were derived from GSE14520. Digits in the blocks represented the Pearson correlation coefficients. Supporting Figure 3: comparison of serum concentrations of 10 potential biomarkers for the first-step validation. Each group included 58 samples. ∗p < 0.05, ∗∗p < 0.01, ∗∗∗p < 0.001, and ∗∗∗∗p < 0.0001. n.s.: not significant. Supporting Figure 4: pairwise correlation analysis of the concentrations between 10 potential biomarkers. Digits in the blocks represented the Pearson correlation coefficients. Supporting Figure 5: pairwise correlation between serum levels of CCL20, LCN2, AFP, and CA199. Data from all samples were used. Digits in the blocks represented the Pearson correlation coefficients. Supporting Table 1: differential expression information and brief description of 16 extracellularly located genes. Supporting Table 2: diagnostic model construction using the comprehensive control (liver cirrhosis+healthy). Supporting Table 3: pairwise comparison of ROC curves. Supporting Table 4: diagnostic model construction using liver cirrhosis as control. [file 7758735.f1.zip › 7758735.f1.pdf]

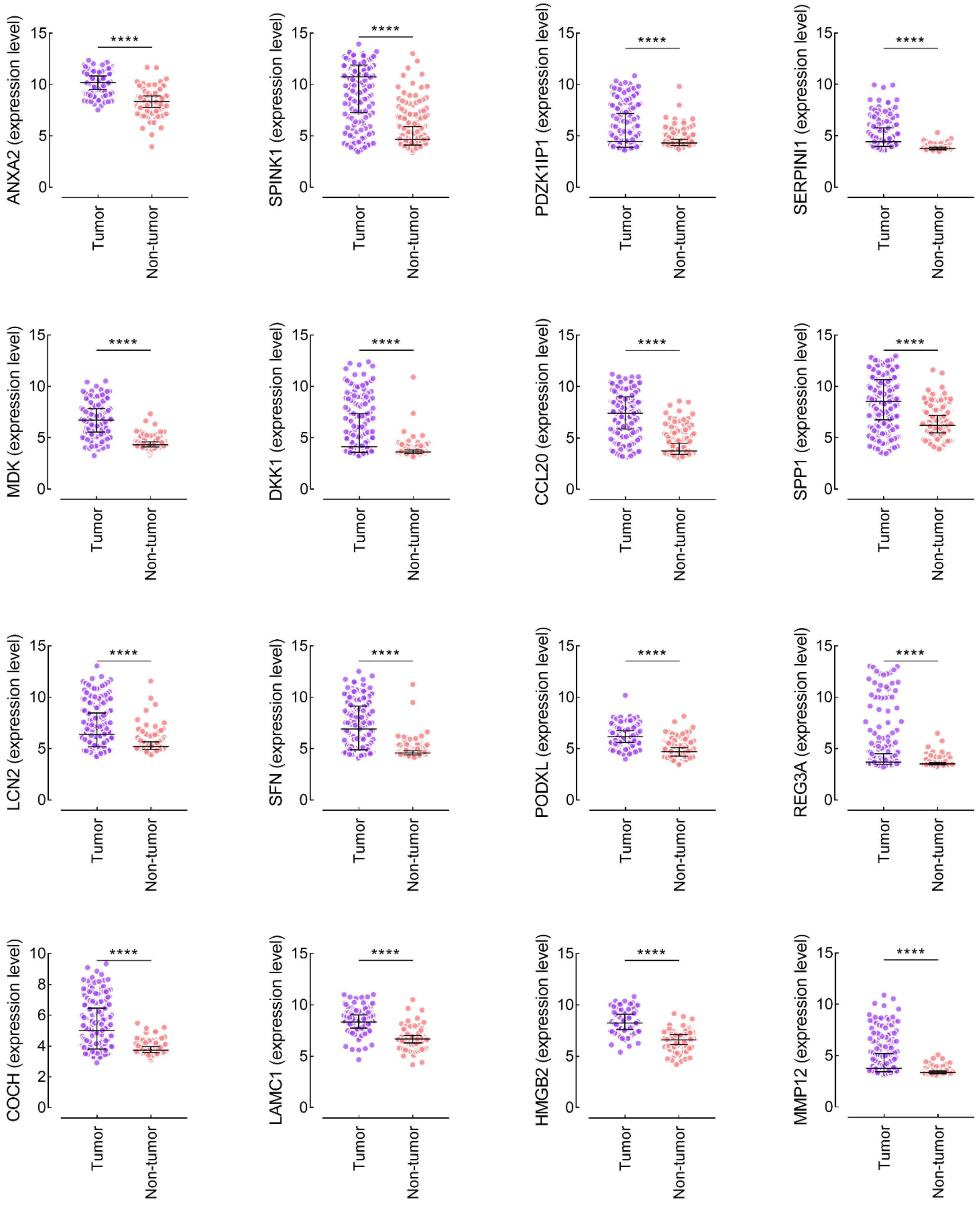

Supporting figure 1. Comparison of expression levels of 16 up-regulated genes, whose protein products were thought to located extracellularly with highest confidence score. Data were derived from GSE14520. ****P<0.0001

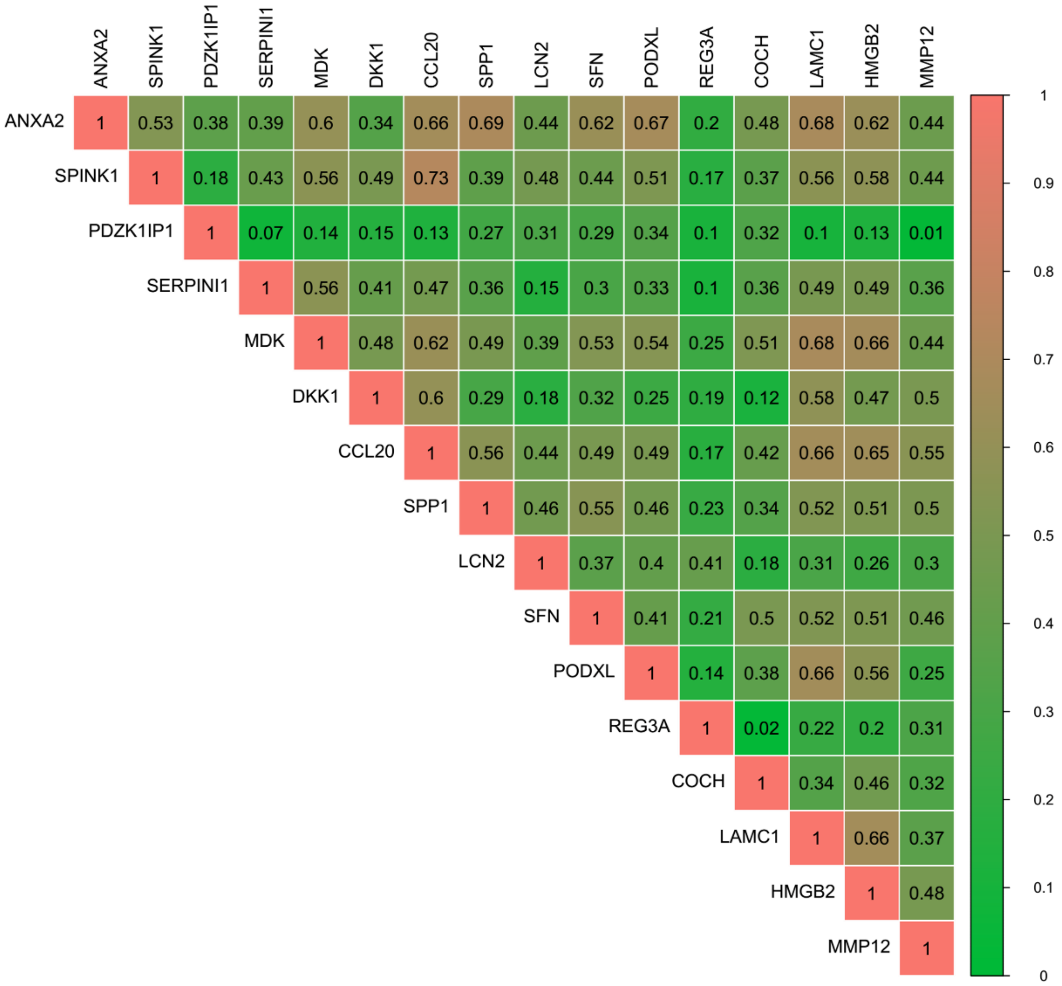

Supporting figure 2. Correlation analysis of expression levels of abovementioned genes. Data were derived from GSE14520. Digits in the blocks represented the person correlation coefficients

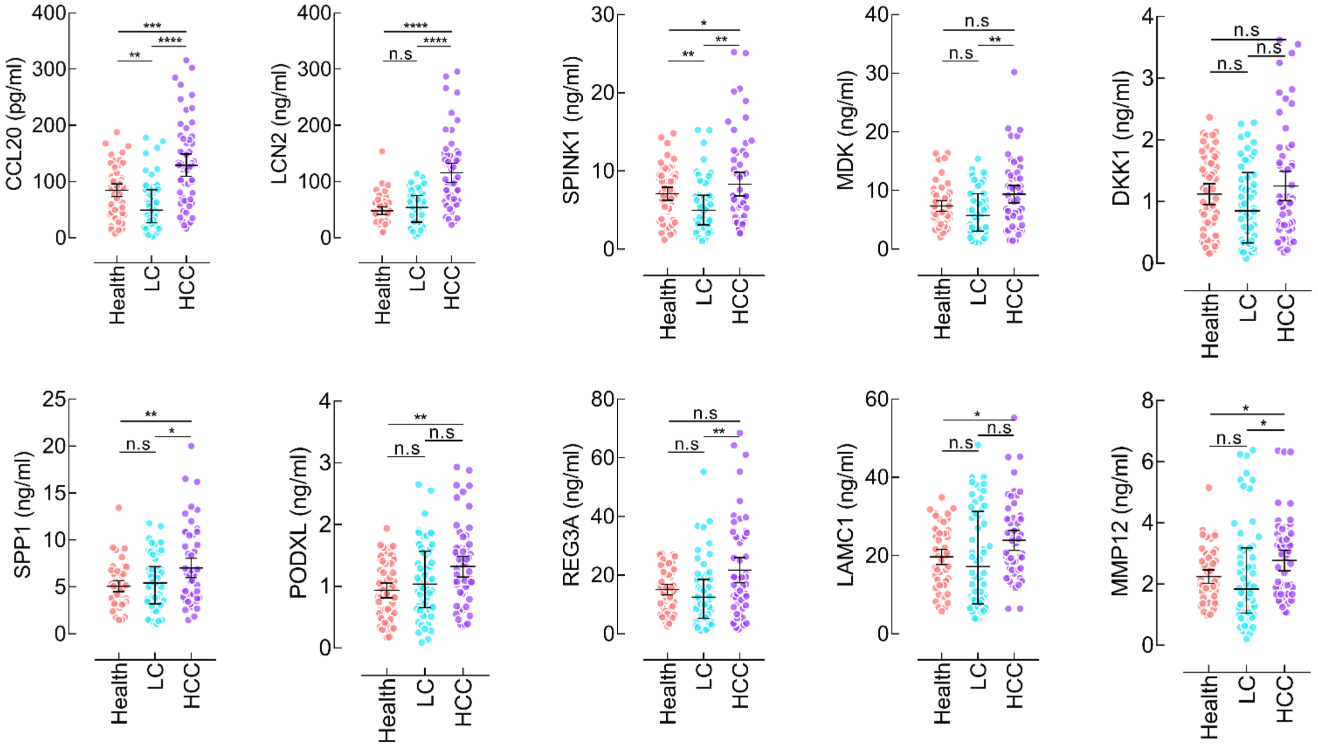

Supporting figure 3. Comparison of serum concentrations of 10 potential biomarkers for the first-step validation. Each group included 58 samples. *P<0.05, **P<0.01,* P<0.001, ****P<0.0001, n.s, no significant.**

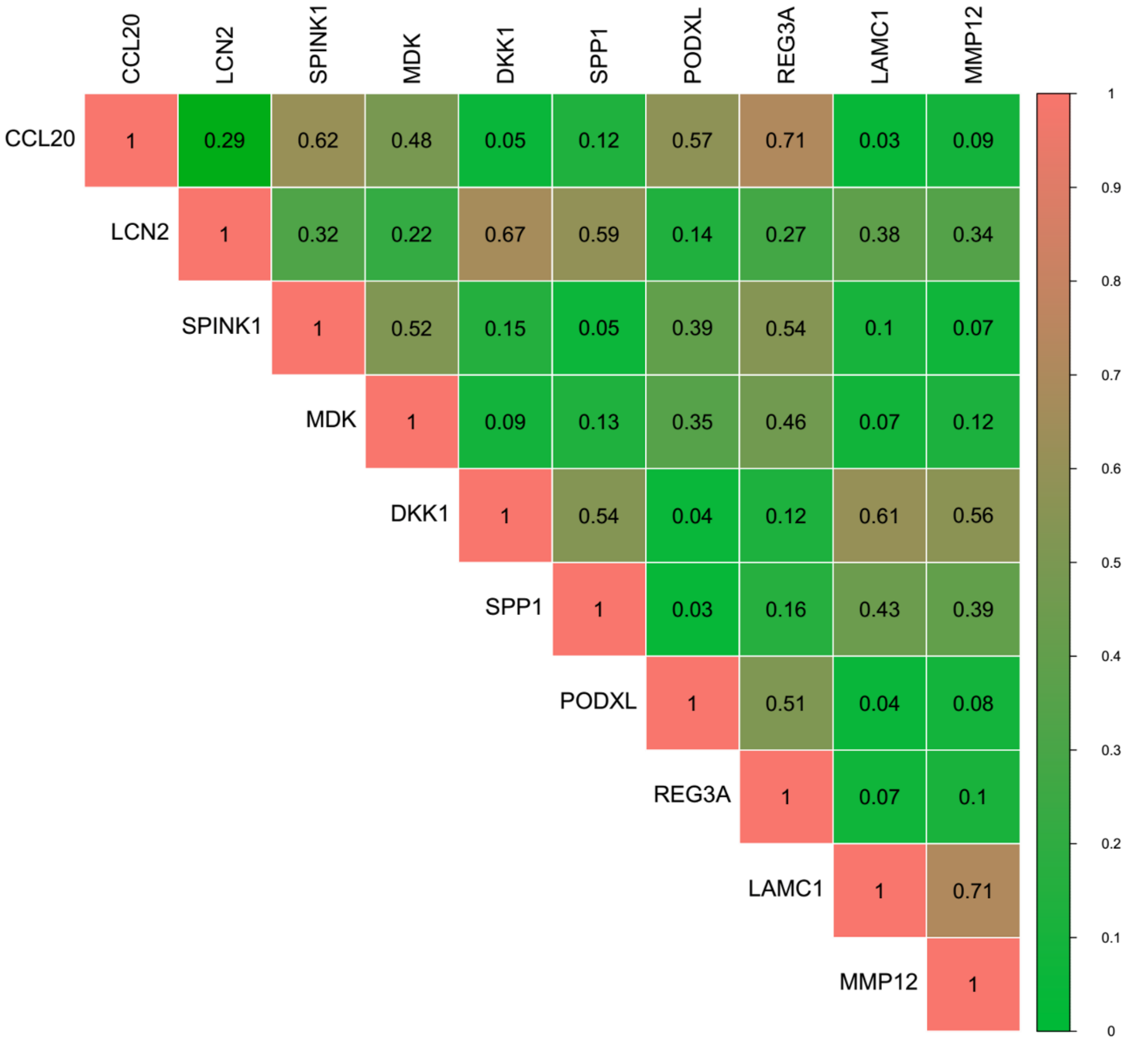

Supporting figure 4. Pairwise correlation analysis of the concentrations between 10 potential biomarkers. Digits in the blocks represented the person correlation coefficients.

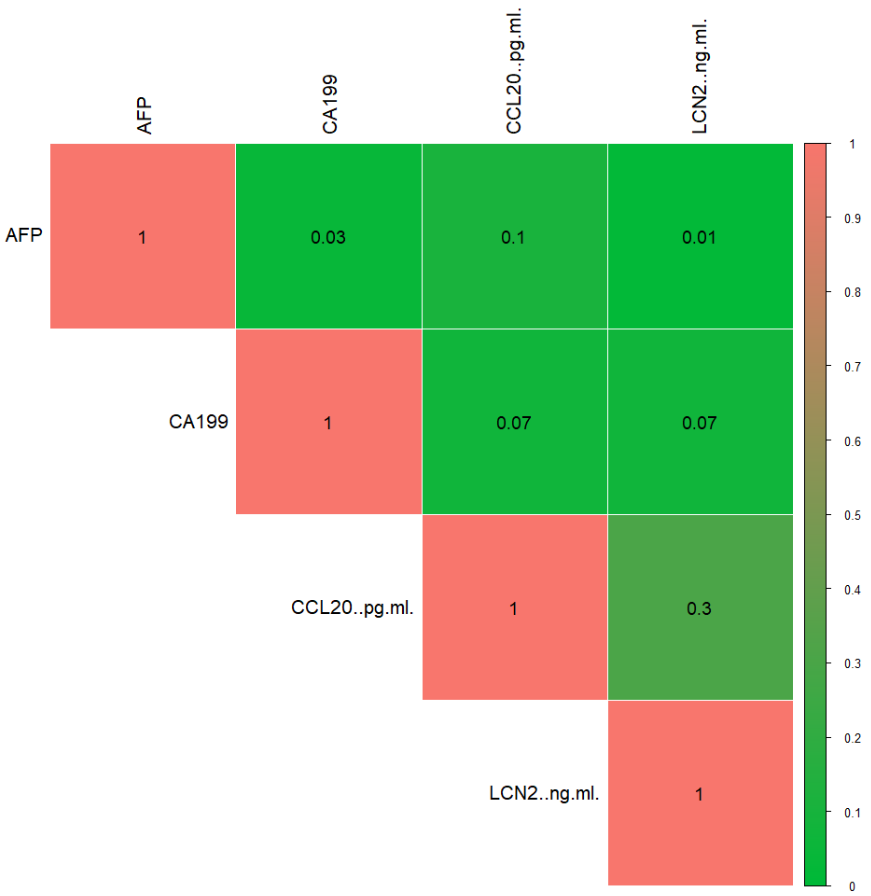

Supporting figure 5. Pairwise correlation between serum levels of CCL20, LCN2, AFP and CA199. Data from all samples were used. Digits in the blocks represented the person correlation coefficients
