# Supplementary material for: Identification of CCL20 and LCN2 as Efficient Serological Tools for Detection of Hepatocellular Carcinoma
Source: Dis Markers. 2022 Mar 10;2022:7758735. doi: 10.1155/2022/7758735 (PMC8930252; doi:10.1155/2022/7758735)
Supplement: Supplementary Materials — Supporting Figure 1: comparison of expression levels of 16 upregulated genes, whose protein products were thought to locate extracellularly with the highest confidence score. Data were derived from GSE14520. ∗∗∗∗p < 0.0001. Supporting Figure 2: correlation analysis of expression levels of abovementioned genes. Data were derived from GSE14520. Digits in the blocks represented the Pearson correlation coefficients. Supporting Figure 3: comparison of serum concentrations of 10 potential biomarkers for the first-step validation. Each group included 58 samples. ∗p < 0.05, ∗∗p < 0.01, ∗∗∗p < 0.001, and ∗∗∗∗p < 0.0001. n.s.: not significant. Supporting Figure 4: pairwise correlation analysis of the concentrations between 10 potential biomarkers. Digits in the blocks represented the Pearson correlation coefficients. Supporting Figure 5: pairwise correlation between serum levels of CCL20, LCN2, AFP, and CA199. Data from all samples were used. Digits in the blocks represented the Pearson correlation coefficients. Supporting Table 1: differential expression information and brief description of 16 extracellularly located genes. Supporting Table 2: diagnostic model construction using the comprehensive control (liver cirrhosis+healthy). Supporting Table 3: pairwise comparison of ROC curves. Supporting Table 4: diagnostic model construction using liver cirrhosis as control. [file 7758735.f1.zip › 7758735.f2.pdf]

Supporting table 1. Differential expression information and brief description of 16 extracellularly-located genes

Name	Log FC			Gene title
	GSE14520	GSE102079	GSE121248	
ANXA2	1.81	1.16	1.16	annexin A2
SPINK1	4.26	3.95	1.04	serine peptidase inhibitor, Kazal type 1
PDZK1IP1	1.06	1.29	1.36	PDZK 1 interacting protein 1
SERPINI1	1.16	1.29	1.21	serpin family I member 1
MDK	2.31	1.55	1.00	midkine (neurite growth-promoting factor 2)
DKK1	1.78	1.84	1.67	dickkopf WNT signaling pathway inhibitor 1
CCL20	3.13	1.51	1.95	C-C motif chemokine ligand 20
SPP1	2.05	2.00	2.10	secreted phosphoprotein 1
LCN2	1.55	1.41	1.84	lipocalin 2
SFN	2.42	2.17	1.44	stratifin
PODXL	1.41	1.51	1.36	podocalyxin-Like Protein 1
REG3A	1.21	1.97	1.96	regenerating family member 3 alpha
COCH	1.45	2.12	1.41	coagulation factor C homolog, cochlin
LAMC1	1.68	1.30	1.14	laminin subunit gamma 1
HMGB2	1.75	1.41	1.08	high mobility group box 2
MMP12	1.21	1.07	1.13	matrix metallopeptidase 12

Brief description (from genecards database, https://www.genecards.org)

ANXA2	plays a role in the regulation of cellular growth and in signal transduction pathways
SPINK1	functions in the prevention of trypsin-catalyzed premature activation
PDZK1IP1	may play an important role in tumor biology
SERPINI1	may play a role in the regulation of axonal growth and the development of synaptic plasticity
MDK	promotes cell growth, migration, and angiogenesis, in particular during tumorigenesis
DKK1	binds to the LRP6 co-receptor and inhibits beta-catenin-dependent Wnt signaling
CCL20	displays chemotactic activity for lymphocytes and repress proliferation of myeloid progenitors
SPP1	be involved in the attachment of osteoclasts to the mineralized bone matrix
LCN2	inhibits bacterial growth as a result of sequestering iron-containing siderophores
SFN	binds to translation and initiation factors and functions as a regulator of mitotic translation
PODXL	be involved in the regulation of both adhesion and cell morphology and cancer progression
REG3A	may be involved in cell proliferation or differentiation
COCH	plays a role in the control of cell shape and motility in the trabecular meshwork
LAMC1	mediate the attachment, migration and organization of cells into tissues during embryonic development
HMGB2	facilitating cooperative interactions between cis-acting proteins by promoting DNA flexibility
MMP12	involved in the breakdown of extracellular matrix in normal physiological processes and disease processes

Supporting table 2 . Diagnostic model construction using the comprehensive control (liver cirrhosis + health)

		B	S.E	Wald	D.F	Sig.	Exp (B), CI 95	Hosmer-Lemeshow	
								χ^2	Sig.
Model_1#	AFP	0.016	0.005	9.718	1	0.002	1.016 (1.006-1.026)	12.086	0.147
	CA199	0.012	0.006	4.288	1	0.038	1.012 (1.001-1.024)		
	Constant	-0.795	0.187	18.139	1	0.000	0.451		
Model_2#	CCL20	0.013	0.003	20.390	1	0.000	1.013 (1.007-1.018)	4.417	0.818
	LCN2	0.045	0.005	80.202	1	0.000	1.046 (1.036-1.057)		
	Constant	-5.309	0.537	97.819	1	0.000	.005		
Model_3#	CCL20	0.013	0.003	20.390	1	0.000	1.013 (1.007-1.018)	4.417	0.818
	LCN2	0.045	0.005	80.202	1	0.000	1.046 (1.036-1.057)		
	Constant	-5.309	0.537	97.819	1	0.000	0.005		

S.E, Standard error. D.F, degree of freedom. Sig., significance. CI 95, 95% confidence interval.

Supporting table 3. Pairwise comparison of ROC curves

AFP	CA199	CCL20	LCN2	Model_1	Model_2
**AFP**	0.0025	0.077	0.0000	0.7542	0.0000
	**CA199**	0.0000	0.0000	0.0000	0.0000
		**CCL20**	0.0000	0.0479	0.0000
			**LCN2**	0.0000	0.0402
				**Model_1**	0.0000
					**Model_2**

Numbers represented the significance levels (P values) of differences between every two AUCs.

Supporting table 4 . Diagnostic model construction using the liver cirrhosis as control

		B	S.E	Wald	D.F	Sig.	Exp (B), CI 95	Hosmer-Lemeshow	
								χ^2	Sig.
Model_2#	CCL20	.014	.003	18.465	1	.000	1.014 (1.005-1.019)	4.785	0.780
	LCN2	.041	.006	52.725	1	.000	1.042 (1.034-1.055)		
	Constant	-4.439	.582	58.142	1	.000	.012		

S.E, Standard error. D.F, degree of freedom. Sig., significance. CI 95, 95% confidence interval.
